# A Rapid Screening Method for Sibutramine Hydrochloride in Natural Herbal Medicines and Dietary Supplements

**DOI:** 10.1155/2021/8889423

**Published:** 2021-08-25

**Authors:** Qi Liang, Yue Zhuang, Jun Ma, Jinyan Wang, Rui Feng, Ruisi He, Zhuoya Luo, Honghao Wang, Ruoting Zhan

**Affiliations:** ^1^Guangdong Institute for Drug Control, Guangzhou, China; ^2^Research Center of Chinese Herbal Resource Science and Engineering, Guangzhou University of Chinese Medicine, Guangzhou, China; ^3^School of Pharmaceutical Sciences, Sun Yat-Sen University, Guangzhou, China; ^4^Market Regulation Administration of Guangdong, Guangzhou, China; ^5^School of Chinese Materia Medica, Ministry of Education, Key Laboratory of Chinese Medicinal Resource from Lingnan, Guangzhou University of Chinese Medicine, Guangzhou, China

## Abstract

Herbal weight loss drugs are becoming more widely used in the fight against obesity, but ineffective regulation of these products have resulted in harmful additives. These products may contain adulterants such as sibutramine hydrochloride that may result in serious adverse health events including death. This work established a color precipitation reaction-based rapid screening method for illegal adulteration of sibutramine hydrochloride in natural herbal medicines (NHM) and dietary supplements (DS). While a variety of chromatography- and electrophoresis-based systems have been reported to measure this analyte, they generally suffer from high costs, complicated sample preparation, and a costly analytical infrastructure. In contrast, we present a simple, handheld kit to assay for sibutramine. The performance metrics of this tool include an average detection time of approximately 3 minutes, which is markedly shorter than conventional methods (HPLC or HPLC-MS, etc.), a detection limit of 0.1 mg per aliquot, and an accuracy of 99.02% (*n* = 820). More strikingly, the sensitivity is 100% (*n* = 278), and the specificity is 98.52% (*n* = 542). The rapid test kit developed from this screening method was evaluated by FDA. In summary, this screening method is a rapid, simple, and low-cost tool for the detection of sibutramine in NHM and DS with superior selectivity and sensitivity. For these reasons, this method is especially suitable for underdeveloped settings because it can be employed onsite without any instrumentation. In addition, this approach could rapidly exclude most of the negative samples to boost efficiency in large-scale samples assay. If necessary, positive samples can undergo further alternate testing methods to confirm the positive results of sibutramine hydrochloride content.

## 1. Introduction

Sibutramine hydrochloride, N-{1-[1-(4-chloroph-enyl)cyclobut-yl]-3-methylbutyl}-N,N-dim-ethylamine hydrochloride monohydrate (C_17_H_26_ClN·HCl·H_2_O) is a reuptake inhibitor of noradrenaline and 5-hydroxytryptamine and has been used as an antiobesity drug since 1997 [1]. It has a molecular weight of 334.33 g/mol.

Sibutramine hydrochloride may cause severe adverse effects including insomnia, psychosis, affective psychosis, panic attacks, delirious state, amnesia, bipolar disorder, hypomanic or manic episodes, and increases the risk of stroke and heart attack [2–15]. Consequently, FDA has withdrawn sibutramine hydrochloride from the US market in October 2010 [16].

Recently, illegal adulteration of natural herbal medicine (NHM) and dietary supplements (DS) with sibutramine hydrochloride has been reported and is dangerous for consumers.

To date, numerous detection methods for sibutramine hydrochloride adulteration in NHM and DS for weight loss have been developed including thin-layer chromatography (TLC) [17, 18], gas chromatography mass spectrometry (GC-MS) [19, 20], liquid chromatography (LC) [21, 22], liquid chromatography-mass spectrometry (LC-MS) [21, 23–27], ion exchange chromatography (IEC) [28], capillary electrophoresis (CE) [29], capillary zone electrophoresis (CZE) [30], nuclear magnetic resonance (NMR) [31], ion mobility spectrometry (IMS) [32], infrared spectroscopy (IR) [33], micellar electrokinetic capillary chromatography (MECC) [34], X-ray powder diffraction (XRPD), and colorimetry [35, 36]. However, there are several disadvantages of these methods including long assay time, expensive instrumentation, and the need for skilled operators. As a result, these methods are an obstacle to consistent and cost-effective testing of NHM and DS.

Color tests have been widely used in drug analysis historically. They are simple, inexpensive, and easy to operate. The results of these methods are easily distinguished and commonly used to assist the identification. Without the precise but unwieldy instruments, the color tests can provide a preliminary estimate of the samples at the various scenes. These results are not as accurate as the instrument analysis, but effective enough for on-site inspection [37–40].

Therefore, this work establishes a rapid, simple, colorimetric screening method for sibutramine hydrochloride in NHM and DS. This method can be developed as a rapid screening kit for onsite analysis of sibutramine hydrochloride in NHM and DS. The method could also be an initial rapid screen prior to instrumental analysis.

## 2. Materials and Methods

### 2.1. Instrumentation

Millipore Milli-Q water purification system (Millipore, USA) and LC-20A HPLC system with UV detector (Shimadzu, Japan) were used.

### 2.2. Reference Standard and Reagents

Sibutramine hydrochloride monohydrate (National Institutes for Food and Drug Control, China; Lot. no. 100624–200401, desiccated for two hours at 105°C before use). Ethyl acetate (AR, Guangzhou Chemical Reagent Co.), phosphoric acid (analytical grade, Guangzhou Chemical Reagent Co.), ammonium reineckate (analytical grade, Shanghai Hengxin Chemical reagent Co., Ltd.), methanol (HPLC grade, Guangzhou Chemical Reagent Co.), triethylamine (analytical grade, Guangzhou Chemical Reagent Co.), acetonitrile (HPLC grade, Merck & Co., Inc., USA), and sodium heptanesulfonate (analytical grade, Shandong Yuwang Industrial Co., Ltd.) were used in this study.

### 2.3. Samples

Zhuoyue Fengzi Slimming Capsule and Fufang Shoubao Capsule were used, courtesy of Guangdong Institute for Drug Control.

### 2.4. Preparation of Ammonium Reineckate Detection Reagent

0.1 g of ammonium reineckate is dissolved in 4 ml of purified water and shaked for 1 min. This solution should be freshly prepared and used within 48 hours.

### 2.5. Rapid Screening Method Procedure

- Powdered sample (0.1 to 0.5 g) was placed in a 7 ml sample vial (A). We then added 3.5 ml of ethyl acetate to vial A, inverted it 5 times, and allowed it to stand for 1 min.
- We used a burette to extract 2 ml of the upper-layer liquid (organic phase) of vial A and transferred it to another sample vial (B). We then added 3 ml of 3% H_3_PO_4_ to vial B. This was inverted 5 times to make a dispersed phase and allowed to stand for 1 min.
- Next, we used a burette to extract 1 ml of the lower-layer liquid (aqueous phase) from vial B and transferred it to a test tube. We then added 1 drop of ammonium reineckate detection reagent. If an obvious light-pink insoluble substance is formed immediately, then the sample is positive for sibutramine. Otherwise, it is negative.

### 2.6. LC Determination of Sibutramine

An LC-20A HPLC system (Shimadzu, Japan) equipped with an automatic sampler and a UV detector was used for the chromatographic analysis. A KQ-300DE ultrasonic generator was purchased from Kunshan Ultrasonic Instruments Co., Ltd., China.

The chromatographic analysis was carried out in isocratic mode on an ODS-2 Hypersil C_18_ analytical column (250 mm × 4.6 mm; 5 *μ*m particle size; Thermo Fisher Scientific, USA). The mobile phase in the HPLC method was methanol-acetonitrile-sodium heptanesulfonate solution (10 : 3 : 7, v/v/v). Sodium heptanesulfonate solution was prepared as follows: add 1000 ml of water in a beaker, dissolve 2.02 g of sodium heptanesulfonate, and then add 0.8 ml of triethylamine and adjust the pH to 3.3 ± 0.1 with glacial acetic acid. The flow rate was 1.0 ml/min, the detection wavelength was 223 nm, and the injection volume was 20 *μ*l.

10 mg of sibutramine hydrochloride was weighed and dissolved in 50 ml of mobile phase. It was then gradually diluted to 0.2 mg/ml in mobile phase with volumetric pipettes and flasks. Quality control solutions were prepared from independent stock standards at 0.5, 1.0, 2.0, 25, 50, 80, and 100 *μ*g/ml in mobile phase. Each quality control solution was filtered through a Sartorius model 0.45 *μ*m polytetrafluoroethylene (PTFE) filter before application in HPLC system. The calibration curve was linear from 0.536 to 107.2 *μ*g/ml (*Y* = 40939*X* + 2517.6, *R*^2^ = 1).

## 3. Results and Discussion

### 3.1. Principle of the Rapid Screening Method

**Figure 1:**
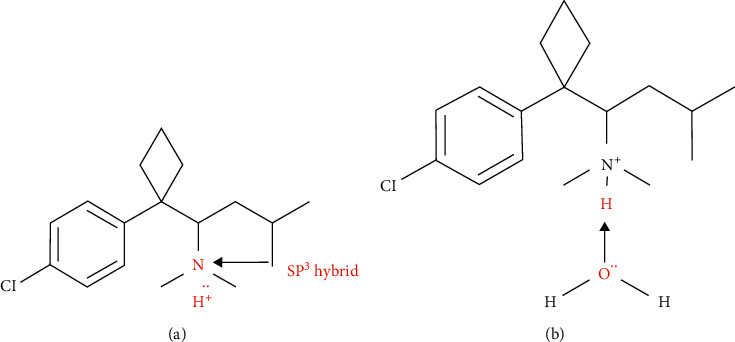
Schematic of dissolution in acid aqueous solution of sibutramine. (a) Protonation of sibutramine. (b) Solvation of sibutramine.

The outermost electron orbital of the nitrogen atom in sibutramine is a SP^3^ hybrid. Thus, there are two lone-paired electrons in the s orbital, which approach the empty orbital of the proton in inorganic acid solutions and form an ammonium cation with proton. As the nitrogen atom combines with high pKa alkyls (pKa_CH4_ = 49), it donates electron part of the positive charge of the ammonium cation that can be dispersed to attain stability. Thus, sibutramine tends to be protonated and well solubilized in acid solution (Figure 1(a)) [41].

Due to the electronegativity of the nitrogen atom in protonated sibutramine, it combines with the nitrogen atom to form a hydrogen bond with water and become solvated (Figure 1(b)). Therefore, sibutramine hydrochloride can easily be dissolved in inorganic acid aqueous solution. On the other hand, as it has a benzene ring and an aliphatic amine structure, sibutramine can also easily dissolve in organic solvents such as methanol, ethanol, *n*-butanol, ethyl acetate, benzene, and dichloromethane.

NHM and DS usually consist of multiple compounds, most of which are colored substances such as phytochromes. Thus, it is necessary to remove these substances before initiating the color reaction for rapid screening because the colored substances may interfere with the colored reaction.

We developed a two-step extraction protocol for sibutramine hydrochloride because of its good solubility in both organic acid solvents and inorganic solution. First, an organic solvent is applied to extract sibutramine hydrochloride from samples, which separates the interfering substances that are insoluble in organic solvents. Second, a liquid-liquid extraction (LLE) is performed with an inorganic acid to further separate sibutramine from the organic solvent that contains the interfering substances.

There are three criteria in selecting the sibutramine hydrochloride extraction buffer: (1) it must dissolve sibutramine hydrochloride; (2) it may not dissolve the inorganic acid solution; and (3) it should be environmentally friendly with no human toxicity. Fortunately, there are numerous organic solvents that meet these criteria including ethyl acetate with good extraction effect and low toxicity.

Both ethyl acetate and sibutramine are electron-donating compounds. They cannot form a hydrogen bond; however, they can attract each other with Van der Waals forces to form weak interactions. Thus, ethyl acetate can easily dissolve and extract sibutramine because they have similar polarities. In addition, ethyl acetate can exclude insoluble substances that may potentially interfere with sibutramine assay.

More importantly, LLE can protonate, solvate, and extract sibutramine and also exclude most competing substances that are insoluble in acid aqueous medium and may interfere with the test results. While many acids could be used, we selected H_3_PO_4_ because of its noncorrodibility, nonvolatility, and convenience.

The sibutramine is protonated on contact with inorganic acid. The polarity of the protonated sibutramine increases, so the difference in polarities between the protonated sibutramine and the ethyl acetate increases, and thus their affinity decreases. Protonated sibutramine then forms hydrogen bonds with water and is solvated and then moves into the inorganic phase from the organic phase.

Sibutramine accepts a proton to form a quaternary ammonium salt (R^+^), and the detection agent should precipitate with the quaternary ammonium salt. For this, we chose ammonium reineckate, which is soluble in water and produces a cation of NH_4_^+^ and an anion of [Cr(NH_3_)_2_(SCN)_4_]^−^.

The cation of R^+^ reacts with the anion of [Cr(NH_3_)_2_(SCN)_4_]^−^ to form an organic salt that is insoluble in acidic aqueous medium. The organic salt is pink, and the precipitate is easily visualized. The ion reaction formula is described in the following equation:

(1) $$\begin{array}{c} R^{+}+{\left[\text{Cr}{\left({\text{NH}}_{3}\right)}_{2}{\left(\text{SCN}\right)}_{4}\right]}^{-}⟶R\left[\text{Cr}{\left({\text{NH}}_{3}\right)}_{2}{\left(\text{SCN}\right)}_{4}\right]↓ \end{array}$$

### 3.2. Optimization of the Method

The main influencing factors of the method were the property and amount of the extraction liquids. To select ethyl acetate and phosphoric acid solution as the extraction liquid has been briefly explained in the above. Based on these results, further optimization of the method was undertaken as follows.

#### 3.2.1. Concentration of Phosphoric Acid Solution

The high efficiency of the extraction step is important because the violent shaking, repeated steps, and long layering time are not always allowed in the rapid screening methods to enhance the performance. This study used dilute phosphoric acid solution and measured the partition coefficient of sibutramine hydrochloride in ethyl acetate and diluted phosphoric acid to optimize the condition.

The partition coefficient was estimated by the following equation:

(2) $$\begin{array}{c} K=\frac{C_{A}}{C_{B}}. \end{array}$$

Here, both *C*_*A*_ and *C*_*B*_ are the concentrations of the test substance in two immiscible solvents while the distribution system becomes balanced.

71.025 mg of sibutramine hydrochloride standard was weighed and transferred into a 200 ml volumetric flask. Then, ethyl acetate was added to dissolve the sample and diluted to the volume. Next, 10.0 ml of the solvent was transferred into a 60 ml separation funnel. This was repeated for 11 more samples. Each held 10.0 ml of diluted phosphoric acid at 0.5%, 1.0%, 3.0%, and 5.0%, respectively. The flask was sealed, shaken, and drained. This was repeated and allowed to stand for layering. We next determined the content of the sibutramine hydrochloride in this two-phase solution with HPLC.

We removed 0.5 ml from the supernatant and the lower layer and placed them in two 10 ml volumetric flasks, respectively. After adding mobile phase to each, we mixed the samples, filtered, and analysed them both with HPLC (Table 1). As shown in Table 1, the concentration variation of the diluted phosphate solution had little effect on the partition coefficients. The coefficients increased slightly with the concentration rising and reached the maximum value when the concentration of the diluted phosphate solution was around 3.0%. Therefore, a 3.0% diluted phosphate solution was chosen as the extraction solution.

#### 3.2.2. Volume of the Extraction Liquid

An orthogonal experiment was performed to optimize the volume of extraction liquids in each step. The concentration of the sample used in this experiment was 0.86 mg/g, which was determined by HPLC at first. The experiment operated by the following steps:

- Powdered sample (0.15 g) was placed in a 10 ml sample vial (A). Then, *V*_1_ ml ethyl acetate was added to vial A, inverted it 5 times, and allowed it to stand for 1 min. Determine the concentration of sibutramine hydrochloride in ethyl acetate (*C*_1_*μ*g/ml) by HPLC.
- *V*_2_ ml of the upper-layer liquid of vial A was transferred into another sample vial (B). *V*_3_ ml 3% H_3_PO_4_ was added to vial B, inverted 5 times to make a dispersed phase and allowed to stand for 1 min. By the way, the amount of *V*_3_ was determined according to a series of fixed ratio of the two phases (*V*_2_ : *V*_3_). Determine the concentration of sibutramine hydrochloride in phosphoric acid solution (*C*_2_*μ*g/ml) by HPLC.

As shown in Table 2, the extraction rates were about 40% when 2 ml ethyl acetate was used. And the rates could be increased to about 80% with 3.5 ml ethyl acetate. Furthermore, double the volume, the rates were increased about only 6 percent, but the concentration of the solution was nearly halved. In the LLE step, the rates were more than 90% in different conditions. It suggests that almost all the sibutramine was extracted into the phosphate acid solution. Considering the final step of the test, high concentration of the solution is favorable for the formation of the precipitate. Thus, the phosphate acid solution should be used as less as possible. However, there is a practical reason, the volume of the liquid used in rapid screening is better to be more than 2 ml for easy operation and observation. Finally, 3.5 ml ethyl acetate and 3 ml phosphate acid solution was considered as the optimal condition.

#### 3.2.3. Effects of Temperature

The effect of temperature on chromic reaction and LLE rate was studied by carrying out the rapid screening method under 10°C, 15°C, 20°C, and 25°C (Table 3). At 10°C, no visible pink precipitates are formed within 30 s. At 15°C or higher temperature, pink precipitates are formed within 30 s. The LLE rates at 10°C, 15°C, 20°C, and 25°C are 99.39%, 99.38%, 99.32%, and 99.23% respectively.

### 3.3. Transfer Efficiency of the Method

By investigating the extraction rate and LLE rate, we demonstrate the transfer efficiency of sibutramine as well as overall feasibility. Extraction rate and LLE rate were determined by HPLC quantification of sibutramine in each extraction step.

The content powder removed from 20 Zhuoyue Fengzi Slimming Capsules was homogenously mixed and weighed. The mean mass was 0.285 g (R.S.D. = 2.76%, *n* = 20). We analytically weighed 0.1 g and added 50 ml of the mobile phase for extraction accompanied with 10 min of treatment in an ultrasonic bath. The supernatant of the extraction solution was filtered through a 0.45 *μ*m PTFE filter before being applied to the HPLC system. The results include that average content of sample is 32.21 mg/g and RSD is 2.58% (*n* = 6); more details are shown in Table 4.

0.1 g of sample was transferred to a 10 ml specimen bottle followed by 8.0 ml of ethyl acetate and inversion 5 times. This was incubated at room temperature for 1 min. We next transferred 3.0 ml of the supernatant to another bottle for the LLE step (solution A). Here, we measured another 2.0 ml of the supernatant and transferred it to a 25 ml volumetric flask. Next, mobile phase was added to dilute to the mark followed by shaking. This was filtered and analyzed with HPLC. The results include that average extraction rate is 78.28% and RSD is 4.33% (*n* = 6); more details are shown in Table 5.

*Procedure*. Solution A was transferred to a 10 ml bottle and mixed with 3.0 ml of 3% phosphoric acid. The sample was inverted 5 times. We moved 1.0 ml of the lower layer into a 10 ml volumetric flask. Mobile phase was added to the mark and then mixed well. We filtered and measured the filtrate with HPLC. This was repeated for five more replicates and results in that average liquid-liquid extraction rate is 59.45% and RSD is 11.01% (*n* = 6); more details are shown in Table 6.

The average transfer efficiency calculated according to the extraction rate and the LLE rate is 46.62% and RSD is 13.63% (*n* = 6); more details are demonstrated in Table 7. It was important to only gently shake the bottle to maintain easy operation, rapid results, and high reproducibility. We also used a shortened laying time. The recommended dosage of sibutramine hydrochloride (10 mg per dose), the content of the determined samples (1.90 mg to 67.02 mg per capsule), and the transfer efficiency (above 30%) give acceptable precision and high detection sensitivity (0.025 mg/ml, equal to 0.112 mg per aliquot, see section LOD). This rapid screening method can determine whether sibutramine hydrochloride is added to NHM or DS products used for weight loss.

### 3.4. Method Validation and Application

The purpose of this rapid screening method is to screen the samples for illegal addition of target analytes within about 3 minutes at the site of inspection. At the same time, dealing with large quantities of samples at low cost was also expected, this method generally consumed about a tenth the cost of conventional analysis methods. One out of it is to quickly screen out whether the sample is a positive one, and the positive rates of such samples are usually about 0∼30% according to the data known. For negative results, no further instrumental analysis is required for confirmatory testing; for positive screening results, a conventional analytical method is used to test the sample to confirm the rapid screening results and accurately detect the content of the target component. The conventional analytical methods mentioned above are high-performance liquid chromatography with reference substances. The screening method has been validated according to Chinese Pharmacopoeia (Version 2010) 9101. These metrics include transfer efficiency, limit of detection (LOD), accuracy, sensitivity, and specificity via HPLC and comparison with the official methods approved by China Food and Drug Administration (CFDA) [42, 43].

#### 3.4.1. LOD

We dissolved sibutramine hydrochloride in ethyl acetate and diluted it with ethyl acetate to obtain a series of concentrations of sibutramine hydrochloride (Table 8). Half of one Fufang Shoubao Capsule was transferred to 3.5 ml of one of the sibutramine hydrochloride standard solutions to a bottle. The Fufang Shoubao product was also tested with an official method and found to be negative for sibutramine and thus was considered to be an interference. Testing was performed as described in the rapid screening method procedure section, and the results are presented in Table 6. When the extraction rate (Table 5) is considered, the LOD in Table 6 will be adjusted based on equation (2). The result is 0.112 mg of sibutramine per aliquot of sample (Table 9):

(3) $$\begin{array}{c} {\text{LOD}}_{\text{adjusted}}=\frac{\text{LOD}\times 3.5}{78.28\%}. \end{array}$$

#### 3.4.2. Method Applications and Average Accuracy, Sensitivity, Specificity, and Selectivity

The rapid screening results can be true positive, false positive, true negative, or false negative versus the official methods approved by CFDA.

Accuracy, sensitivity, and specificity are the important parameters that characterized the precision of this method. These three parameters were calculated by the following equations, respectively:

(4) $$\begin{array}{c} \text{accuracy}=\frac{\left(S-S_{F}\right)}{S}\times 100\%, \end{array}$$

(5) $$\begin{array}{c} \text{sensitivity}=\frac{\left(P-N_{F}\right)}{P}\times 100\%, \end{array}$$

(6) $$\begin{array}{c} \text{specificity}=\frac{\left(N-P_{F}\right)}{N}\times 100\%. \end{array}$$

Here, *S* is the total number of test specimens, *P*_*T*_ is the number of the true positive specimens, *N*_*T*_ is the number of the true negative specimens, *N*_*F*_ is the number of the false negative specimens, *P*_*F*_ is the number of the false positive specimens, *S*_*F*_ is the summation of *P*_*F*_ and *N*_*F*_, *P* is the summation of *P*_*T*_ and *N*_*F*_, and *N* is the summation of *N*_*T*_ and *P*_*F*_.

Based on laboratory, civic, and provincial applications by different experimental level operators, the performance of the method is valuated below.

In our laboratory, we studied 60 batches of proprietary Chinese medicines and health foods that claimed an antiobesity function. These were purchased from three different cities. We compared the rapid screening results to the CFDA-approved method; the findings were completely consistent except for one false positive result (lab test results in Table 10).

With the assistance of two civic institutes of drug control, 47 batches of proprietary Chinese medicines and health food sold for weight loss were purchased and examined by technicians at the two institutes. The results via the rapid screening method concurred with the CFDA-approved methods (small-scale results in Table 10).

In August 2008, the Guangdong province FDA launched a joint operation on antiobesity health food supervision in which 238 dealers were sampled, and 713 batches of weight loss DS were tested by the local FDA inspectors in the province who have a little experiment training. Two hundred and sixty-six brands of antiobesity health foods sold. Compared with the CFDA-approved method, only 7 out of the 713 samples were false positive by the rapid method (full-scale results in Table 10).

**Figure 2:**
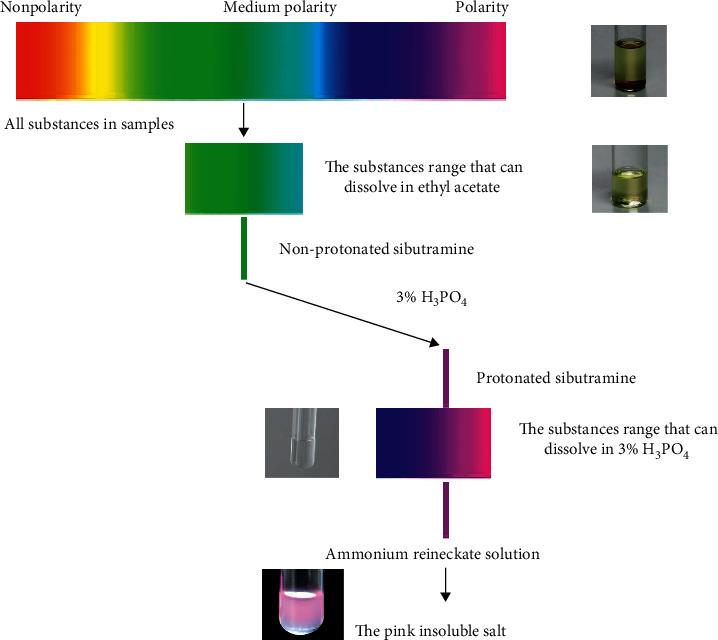
General schematic of the rapid screening method.

In this method, the target compound undergoes protonation, LLE, and a precipitation reaction (Figure 2). Each step offers selectivity and excludes interference substances. Therefore, the method produces high-accuracy results as shown above.

### 3.5. Verification and Evaluation from FDA

We requested the FDA division of pharmaceutical analysis to evaluate the rapid test kit in 2011. They prepared standards to estimate the detection limit of this test by spiking dietary supplements with sibutramine. They found that they could visually identify spiked dietary supplements with as little as 0.1 mg of sibutramine. That is significant because a typical dose of sibutramine is 5 mg, so this detection limit is 50 times lower than the expected level of sibutramine in adulterated dietary supplements. They concluded that the rapid test kits are extremely useful to screen dietary supplements for the presence of sibutramine.

## 4. Conclusions

This study describes a new and easy on-site inspection method with high specificity, fast assay time, and low cost. This tool is likely to be used widely by local Food and Drug Administration personnel. The assay may overcome the shortcomings of current technology and solve the difficulties encountered in the supervision and administration of food and drugs to ensure public health safety.

This study uses a fast and novel colorimetric reaction. The methodology, choice of the reagent and test solution, evaluation model, LOD values, and verification steps described here are novel and may be useful to other researchers in the field.

While this rapid screening test is simple and easy to use, it does require a skilled operator and could not be used by common consumers without training. Thus, we are working on an improved version that can be employed by unskilled operators.

## Figures and Tables

**Table 1:** The results of partition coefficients.

System^*∗*^	Sample	Peak area	*C*^A^ or *C*^B^ (*μ*g/ml)	Partition coefficients *K*	*K* (avg.)	R.S.D. % (*n* = 3)
1	B	13958213	340.89	36.62	36.72	1.31
	A	383660	9.31			
	B	13936925	340.37	36.29		
	A	386525	9.38			
	B	14138345	345.29	37.24		
	A	382022	9.27			

2	B	13961488	340.97	42.00	40.37	3.51
	A	334942	8.12			
	B	14056876	343.30	39.64		
	A	357049	8.66			
	B	14024944	342.52	39.47		
	A	357868	8.68			

3	B	14275491	348.64	45.18	46.70	3.18
	A	318567	7.72			
	B	14247243	347.95	48.14		
	A	298507	7.23			
	B	14171506	346.10	46.77		
	A	305466	7.40			

4	B	14118285	344.80	46.67	47.04	2.78
	A	305057	7.39			
	B	14198116	346.75	48.50		
	A	295231	7.15			
	B	14093721	344.20	45.96		
	A	309151	7.49			

^*∗*^System 1: ethyl acetate/0.5% phosphoric acid; 2: ethyl acetate/1.0% phosphoric acid; 3: ethyl acetate/3.0% phosphoric acid; 4: ethyl acetate/5.0% phosphoric acid; ^A^*C*: the organic phase; and ^B^*C*: the aqueous phase.

**Table 2:** The analysis results of the orthogonal experiment.

No.	*V*_1_ (ml)	*C*_1_ (*μ*g/ml)	Extraction rate (%)	*V*_2_ (ml)	*V*_2_ : *V*_3_	*V*_3_ (ml)	*C*_2_ (*μ*g/ml)	LLE rate (%)	Transfer efficiency (%)
1	7	16.59	87.74	4.0	1 : 3	12.0	5.36	99.03	86.89
2					2 : 3	6.0	10.63	94.94	83.30
3					4 : 3	3.0	18.88	91.27	80.08

4	3.5	30.91	81.67	2.0	1 : 3	6.0	9.97	95.80	78.24
5					2 : 3	3.0	20.23	104.64	85.46
6					4 : 3	1.5	41.00	103.17	84.26

7	2.0	29.84	43.30	1.0	1 : 3	3.0	17.84	179.33	77.65
8					2 : 3	1.5	23.85	119.90	51.92
9					4 : 3	0.75	48.85	122.78	53.16

*C*: the concentration of sibutramine hydrochloride in the detection solution. LLE rate: the extraction rate of liquid-liquid extraction step. Transfer efficiency: the efficiency of the sibutramine transfer from the sample to the test liquid.

**Table 3:** Effect of temperature on colorimetric reaction.

Temperature (°C)	Phenomena
10	No obvious pink insoluble substance forms within 30 s
15	Pink insoluble substance forms within 30 s
20	Pink insoluble substance forms within 30 s
25	Pink insoluble substance forms within 30 s

**Table 4:** The results of determination of the samples.

Sample weight (g)	Peak area A	*C*^1^ (*μ*g/ml)	*X*^2^ (mg/g)	Average (mg/g)	R.S.D. % (*n* = 6)
0.10685	2828469	69.03	32.30	32.21	2.58
0.10835	2848987	69.53	32.09		
0.10627	2737640	66.81	31.43		
0.10711	2748151	67.07	31.31		
0.10810	2884583	70.40	32.56		
0.10192	2805760	68.47	33.59		

^1^*C*: the concentration of sibutramine hydrochloride in the detection solution, same as below. ^2^*X*: the content of sibutramine hydrochloride in the test specimen.

**Table 5:** Results of the extraction.

Sample weight (g)	Peak area A	*C* (*μ*g/ml)	Extraction rate (%)	Average (%)	R.S.D. % (*n* = 6)
0.10281	1045088	25.47	76.90	78.28	4.33
0.10362	1031081	25.12	75.28		
0.10281	1067915	26.02	78.59		
0.10288	1042179	25.40	76.64		
0.10428	1169179	28.50	84.84		
0.10126	1036531	25.26	77.44		

**Table 6:** Results of liquid-liquid extaction rate.

Content in solution A (mg)	Peak area A	*C* (*μ*g/ml)	LLE rate (%)	Average (%)	R.S.D. % (*n* = 6)
0.9550	622675	15.15	47.59	59.45	11.01
0.9422	799286	19.46	61.97		
0.9759	768581	18.71	57.52		
0.9523	779353	18.98	59.78		
1.0687	968675	23.60	66.25		
0.9472	824669	20.08	63.61		

**Table 7:** Calculation of transfer efficiency. The transfer efficiency is the product of the extraction rate and the LLE rate.

No.	Extraction rate (%)	LLE rate (%)	Transfer efficiency (%)	Average (%)	R.S.D. % (*n* = 6)
1	76.90	47.59	36.59	46.62	13.63
2	75.28	61.97	46.65		
3	78.59	57.52	45.21		
4	76.64	59.78	45.81		
5	84.84	66.25	56.21		
6	77.44	63.61	49.26		

**Table 8:** The determination of detection limit.

Sample	Concentration of sibutramin hydrochloride (mg/ml)	Precipitate formed
1	0	No
2	0.008	No
3	0.010	No
4	0.012	Not sure
5	0.025	Yes
6	0.050	Yes
7	0.100	Yes

When testing sibutramine within a dietary supplement, it was determined that 0.025 mg/ml sibutramine hydrochloride was the lowest visually detectable limit.

**Table 9:** The determination of adjusted detection limit.

Sample	Content of sibutramin hydrochloride (mg per aliquot^*∗*^)	Precipitate formed
1	0	No
2	0.036	No
3	0.045	No
4	0.054	Not sure
5	0.112	Yes
6	0.224	Yes
7	0.447	Yes

According to the results listed in Table 9, the LOD is 0.112 mg of sibutramine hydrochloride per aliquot for this method. The method is simple and rational. Aliquot: a certain amount of sample may vary from 0.1 to 0.5 g.

**Table 10:** The primary technical parameters of this method.

	*P*_*T*_	*N*_*T*_	*P*_*F*_	*N*_*F*_	Accuracy (%)	Sensitivity (%)	Specificity (%)
Lab test (*S* = 60)	36	23	1	0	98.33	100	95.83
Small-scale (*S* = 47)	34	13	0	0	100	100	100
Full-scale (*S* = 713)	208	498	7	0	99.02	100	98.61
Total (*S* = 820)	278	534	8	0	99.02	100	98.52

## Data Availability

The data used to support the findings of this study are available from the corresponding author upon request.
